# Criminal and Obscene Quack Literature

**Published:** 1859-01

**Authors:** 


### *Criminal and Obscene Quack Literature.*

It is high time that the laws of the land against the publication of obscene works was enforced against a class of quack productions with which our community is flooded, and which has found its way into the columns of our newspapers in the form of advertisements. In many of our cheap daily and weekly newspapers, may be found advertisements of books, addressed especially to females, which—we speak advisedly—are quite as much calculated to corrupt the morals of the young of both sexes, as are any of the acknowledged, and too well known, obscene publications, against the circulation of which stringent laws are passed—though we fear but seldom executed. We remember on one occasion to have found, nicely tucked away in the dish closet of a most respectable married lady, a work which she had obtained in reply to one of these specious quack advertisements, a book which we know she would have been ashamed to have had her husband discover her perusing. And it is not married ladies alone that procure and read these works, as many a physician has probably the opportunity of discovering. A prurient curiosity leads many a young woman to reply to these advertisements, and procure works which "open the eyes" of the possessor of them.

But books are not the only objectionable form in which indecent quack literature, meets the eyes of the female sex. The mails are burdened with circulars, and the family newspaper with open, undisguised advertisements of obscene character, AND CRIMINAL INTENT. We appeal to the record.

We have before us a circular which has come into our possession, which enters into a long and labored disquisition on the virtues of a medicine, the intent of which is to *prevent pregnancy!* The wife is informed that she can use the medicine without the knowledge of her husband or most intimate friends, and by inference at least, the unmarried, that she may indulge in the most unbounded licentiousness without being detected, provided only she has this medicine at hand—thus attempting to defraud the husband of his offspring, and to demoralize young women.

But these circulars are *secret* missives, and their influence is somewhat limited. There is another class of immoral and indecent publications found in advertisements in the family newspaper, and even the *religious* press of the country is not entirely free from contamination. We have before us a weekly newspaper published in one of the "most straightest" of the New England States, in which may be counted *twenty-eight* different quack advertisements, (not including hair tonics,) occupying *nearly half* of the advertising department of the paper, and comprising nearly one-fourth of its whole contents! *Ten* of these advertisements are embellished with cuts, several of which are supposed to represent the "wondrous wise" head which originated the nostrum advertised. *Fifteen*, that is more than half of these advertisements, are especially addressed to those having "secret diseases," and to females, and THREE of them hold out the caution (the *intent* is perfectly transparent!) that married ladies should not use at all, or at least during the three first months of pregnancy, the nostrum advertised; while a FOURTH unblushingly offers to send for three dollars "*the only safe and sure preventive from pregnancy.*" Several of the advertisers proffer private treatises on disease, embellished with from one to two hundred plates, some of them colored!

In conclusion, and without any further comment, we copy, as nearly as we can, one of the advertisements, as a type of the rest, omitting name and address. We choose this for its brevity.

